# Reporter Group-Labeled Synthetic Cellulose: Structural Characterization and Utilization in Mapping the Cellulose Chain-Cleavage Modes of Cellulases

**DOI:** 10.1021/acs.biomac.5c02515

**Published:** 2026-02-25

**Authors:** Gaurav Singh Kaira, Manuel Eibinger, Chao Zhong, Bernd Nidetzky

**Affiliations:** † Institute of Biotechnology and Biochemical Engineering, Graz University of Technology, NAWI Graz, Graz A-8010, Austria; ‡ Austrian Centre of Industrial Biotechnology (Acib), Graz A-8010, Austria

## Abstract

Iterative β-1,4-glucosylation of p-nitrophenyl (pNP)-β-cellobioside by cellodextrin phosphorylase was applied to synthesize reducing-end reporter group-labeled cellooligosaccharides (average degree of polymerization, DP: ∼5.7; DP range: 4–10) which, upon self-assembly in solution, precipitate as a cellulose allomorph II crystalline material. Atomic force microscopy revealed that the synthetic cellulose-pNP forms nanoscale sheet-like assemblies, with thickness ∼4 nm. The labeled cellulose was used to characterize the chain cleavage specificities of cellulose-degrading enzymes. Reactions were monitored by the release of soluble products as well as the change in oligosaccharide composition of the residual solid material, measured by mass spectrometry. The products formed by hydrolase systems (*Trichoderma reesei* cellulases; *Clostridium thermocellum* cellulosome) are controlled by processive chain cleavages from the chain ends. Atomic force microscopy shows that *T. reesei* Cel7A preferentially attacked sheet edges, with degradation efficiency and directionality dependent on local nanoscale structure. Collectively, we show synthesis of reporter group-labeled cellulose for probing enzymatic chain depolymerization activity on a crystalline solid substrate.

## Graphical abstract

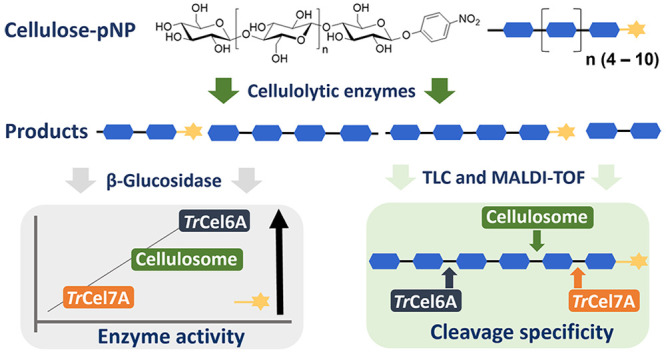

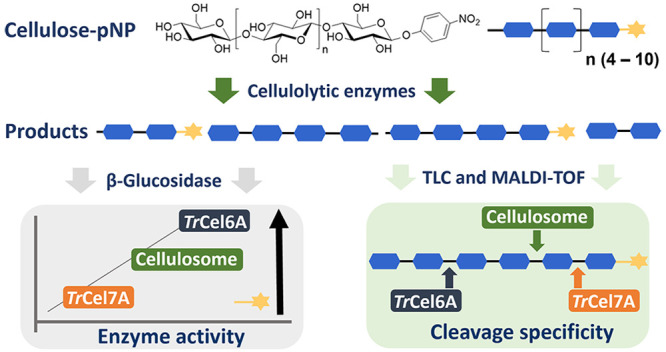

## 1 Introduction

Enzymatic depolymerization of polymeric materials is central to sustainable circular-economy strategies and underpins both lignocellulosic biorefineries and emerging enzymatic recycling of synthetic polymers (“plastics”).^1–3^ Because polymers are usually insoluble in water, their biological depolymerization relies on enzymes that act on solid substrates, making the interfacial nature of the degradation process challenging to characterize biochemically and mechanistically.^4^ Water-soluble model substrates are therefore often used as polymer surrogates in the study of polymer-degrading enzymes.^5–10^ However, these model substrates lack key material features such as chain organization into solid-state exhibiting some degree of structural ordering, therefore enzyme behavior in homogeneous solution may not reflect activity on native-like substrates.^5,9,11^

Enzymatic depolymerization can involve chain-end (exo) cleavage, internal (endo) cleavage, or both at a time.^6,12^ Polymer degradation may also proceed processively, where an enzyme performs multiple cuts on the same chain before acting on another.^12,13^ Although processivity is a well-known feature of exocleaving enzymes, it can also occur in enzymes that catalyze endolytic cleavage.^12,13^ To map the chain cleavage mode of the enzymes, one of the original chain ends must remain identifiable as the polymer chain is cleaved into smaller fragments.^14–16^ This is commonly achieved by labeling a chain-end of the polymer with a reporter group.^15,17^ Polymer substrates tailored to an advanced characterization of polymer-degrading enzymes are therefore expected to exhibit the following characteristics: well-defined and experimentally tractable degree of polymerization (DP) of the polymer chain(s); reporter group label at one chain end; and polymer chain assembly into a solid material of representative (e.g., native-like) structural organization. Moreover, such solid substrates are compatible with complementary analytical techniques that allow the investigation of morphological and molecular-level features.^18,19^ Atomic force microscopy (AFM) provides nanoscale spatial resolution and force-sensing capabilities that enable direct visualization of surface topography, enzyme–substrate interactions, and degradation dynamics under near-physiological conditions.^20,21^

Hydrolytically cellulose chain-cleaving enzymes (cellulases) show activity with soluble model substrates, as reported in numerous earlier studies including the seminal works of Claeyssens and Biely.^14,16,22,23^ However, efficient catalysis by cellulases generally requires adsorption to a solid cellulose surface, where the compact substrate structure loosens to enhance molecular disorder and expose cellulose chains.^24^ This emphasizes the importance of preparative access to suitably labeled cellulose materials for enzyme characterization. Reporter groups in cellulase model substrates are usually installed at the reducing end of the cellulose chain. Reasons are the facile chemical manipulation of the reducing chain end and the broad tolerance of cellulases to the chemical structure of reporter group at that position.^15,25^ Common reporter groups are chromophores (e.g., p-nitrophenyl, 2-chloro-4-nitrophenyl)^5,6,14^ and fluorophores (e.g., methylumbelliferyl, anthranilic acid).^5,6,14,15,23,25^ Radiolabeling^16,17,22^ and reduction of the reducing chain end^15,16,25^ have also been used. Derivatization of the non-reducing chain end, by contrast, usually disrupts the chain-end recognition by the relevant chain end-cleaving exocellulases. For example, 4,6-*O*-(3-ketobutylidene)-4-nitrophenyl-β-d-cellopentaoside is a commercial model substrate of cellulases that involves a 3,4-ketal group at the nonreducing end^26^ and so excludes the activity of the non-reducing end-targeting cellobiohydrolases, such as *T. reesei* Cel6A.

Traditionally, labeled celluloses are prepared by derivatizing purified solid cellulose (e.g., Avicel microcrystalline cellulose).^15,25^ These materials typically involve extensive mechanical and chemical processing and are never truly native regardless of top-down or bottom-up preparation. However, labeling cellulose faces limitations due to the incomplete derivatization of reducing chain-ends^15,25^ and the high degree of polymerization of the cellulose (e.g., DP 150–500 in Avicel^6^), which is too large for precise analysis of enzymatic cleavage patterns.

Bottom-up synthesis of cellulose chains by iterative β-1,4-glucosylation of a reporter group-labeled β-glucoside substrate (“primer”) thus represents an interesting alternative. Cellodextrin phosphorylase (CdP; EC 2.4.1.49; GH94 ^27,28^ ) catalyzes synthesis of short cellulose chains (DP ≥ 7), that spontaneously form cellulose II crystalline material.^29^ A broad range of primer modifications (e.g., alkylalcohols, azides, and polyethylenglycols) can be incorporated at the reducing end, exploiting the wide tolerance of CdP for the structure of the β-glucoside primer.^29,30^ Recently, reducing-end p-nitrophenol-labeled cello-oligosaccharides synthesized by *C. thermocellum* CdP were shown to self-assemble into cellulose II^19,31^ but these modified materials were not further evaluated for enzyme-characterization studies.

Here, we report reducing-end 4-nitrophenol-labeled cellulose (cellulose-pNP) synthesized by CdP from *Clostridium cellulosi*. The labeled cellulose involves chains of DP 4–10 and is of high cellulose II crystallinity, consistent with recent findings.^19,31^ We used the cellulose-pNP for activity characterization and chain cleavage mode determination for different hydrolytic enzymes. Using cellulases and a cellulosome, we show that cellulose-pNP enables clear differentiation of enzymatic chain-cleavage modes. Real-time atomic force microscopy of *Tr*Cel7A degrading cellulose-pNP revealed that nanoscale sheet edges serve as accessible attack sites, while local variations in nanoscale packing modulate both its efficiency and directionality.

## 2 Experimental Section

### 2.1 Materials

β-Glucosidase, endoglucanase (from *Trichoderma longibrachiatum*; *Tl*Cel7B), and cello-oligosaccharides (G2-G5) were obtained from Megazyme (Dublin, Ireland). Avicel PH-101, α-d-glucose 1-phosphate disodium salt hydrate, and pNP-G were obtained from Sigma-Aldrich (St. Louis, MO). p-Nitrophenyl β-d-cellobioside (pNP-G2) was obtained from Carbosynth (Compton, U.K.). Unless stated, all chemicals were of the highest grade available from Carl Roth + Co KG (Karlsruhe, Germany).

### 2.2 Enzymes

#### 2.2.1 Cellodextrin Phosphorylase

The CdP from *C. cellulosi* was used.^32^ Stocks of purified enzyme (∼20 mg/mL) in 50 mM MES buffer, pH 7.0, were stable at −20 °C for several weeks. The standard activity assay^32^ was done at 45 °C and pH 7.0 using α-glucose 1-phosphate and G2 (50 mM each). Phosphate release was measured in samples taken at certain times.^32^ Alternative substrates tested in place of G2 were pNP-G1 and pNP-G2, which were also used at 50 mM.

#### 2.2.2 Cellulases and Cellulosomes

*T. reesei* cellulases were used. The native cellulase mixture was prepared from the clear culture supernatant of *T. reesei* strain SVG17, as described in earlier work.^33^ The enzyme was buffered to 50 mM sodium acetate, pH 5.0, supplemented with 0.05% (w/v) sodium azide. It was diluted to a working stock of 0.25 mg/mL and stored at 4 °C for ∼10 months without loss of protein and activity. The *T. reseei* cellobiohydrolase I (*Tr*Cel7A) was purified from the cellulase preparation as reported elsewhere.^20^ The *T. reseei* cellobiohydrolase II (*Tr*Cel6A) was expressed in *Komagataella phaffii*.^34^ It was lyophilized and stored at −20 °C until further use. The *Tr*Cel7A and *Tr*Cel6A were brought to 50 mM sodium acetate buffer, pH 5.0, and were diluted to a stock concentration of 0.25 mg/mL. Stored at 4 °C, the enzymes were stable for ∼3 months.

The cellulosome from *C. thermocellum* ATTC 27405 was used. Purified cellulosome was obtained by reported methods.^20^ The enzyme preparation was kept at 0.14 mg/mL in 30 mM MOPS buffer, pH 7.0, containing 100 mM NaCl and 10 mM CaCl_2_. Stored at 4 °C, the cellulosome stock was stable (protein and activity) for ∼10 months. Protocol from literature^20^ was used to release the enzymatic subunits of the cellulosome from the scaffold protein. The preparation (free enzymatic subunits with scaffold protein removed) refers to a disassembled cellulosome. Stored at 4 °C and at a concentration of 0.10 mg/mL in the same buffer as that above for the cellulosome, it was stable for ∼6 months.

### 2.3 Substrate Synthesis

Reactions (in 1.0 mL total volume) were performed at 45 °C and an agitation rate of 300 rpm using a ThermoMixer C (Eppendorf, Vienna, Austria). The reaction mixture contained 100 mM α-glucose 1-phosphate, 10 mM pNP-G2 or G2 (for unlabeled cellulose), and 1.0 U/mL purified CdP in 50 mM MES buffer, pH 7.0. Synthesis reactions after 6 h yielded insoluble material as a white precipitate. The insoluble material was centrifuged off (21130 *g*, 5 min, 25 °C; Eppendorf 5424 R, Hamburg, Germany), washed three times with Milli-Q water and was stored wet at 4 °C. Approximately 0.5 g wet material was lyophilized for further analysis. In addition, the supernatant of the reaction mixture was heated (95 °C, 5 min) to inactivate the enzyme and was centrifuged. The conversion of α-glucose 1-phosphate was determined by the phosphate released into the supernatant, through a colorimetric assay.^32^ The mole% yield of cellulose-pNP is expressed as cellulose-pNP synthesized [mM] per α-glucose 1-phosphate consumed [mM]. The concentrations of unlabeled cellulose and cellulose-pNP were measured gravimetrically (*n* = 3). The soluble pNP-labeled oligosaccharides were synthesized under similar conditions but with a higher acceptor concentration (25 mM pNP-G2), and reduced time (2.5 h). Additionally, the preparation was treated with *Escherichia coli* α-glucose 1-phosphate phosphatase^35^ (20 μg/mL, at 37 °C, 300 rpm for 1 h in 50 mM MES buffer, pH 7.0) and centrifuged (16,900 *g*, 10 min, 4 °C; Eppendorf 5418 R) to remove the solids. The soluble preparation was stored at 4 °C and used as a standard (Figure S1).

### 2.4 Substrate Analytical Characterization

#### 2.4.1 Matrix-Assisted Laser Desorption Ionization Time-of-Flight Mass Spectrometry (MALDI-TOF MS)

The cellulose-pNP suspended in water (∼5 mg/mL) was measured according to a reported method.^36^ Mass spectra were analyzed using mMass (http://www.mmass.org/). The number-average molecular mass (*M̅*_n_) was calculated using the relationship, *M̅*_n_ = ∑*_i_* (*N_i_* × *M_i_*)/∑*_i_* *N_i_*, where *N_i_* is the peak intensity of the *i-*th cello-oligomer species and *M_i_* is the molar mass of that species. Here, the *N_i_* presents the total intensity of a cluster of peaks derived from each cello-oligomer species during measurement. The average DP is calculated using the relationship, DP = (*M̅*_n_ – 139)/*M*_o_, where *M*_o_ is the molecular mass of dehydrated glucose in cellulose (162.2 Da).^36^ The residual solid material after incubation with cellulolytic enzyme was analyzed, as described above. The relative percentage (%) of pNP-oligosaccharides in the sample was determined with the relationship, *RP*_labeled_ = ∑*_i_* *I*_*i*_/(∑*_i_* *I*_*i*_ + ∑*_i_* *I’*_*i*_)•100, where *I*_i_ is the total peak intensity of the *i*-th pNP-labeled cello-oligomer species and *I’*_*i*_ presents the peak intensity of the *i*-th unlabeled cello-oligomer species.

#### 2.4.2 Proton Nuclear Magnetic Resonance (^1^H NMR)

The ^1^H NMR spectra of the lyophilized cellulose-pNP dissolved in 4% NaO^2^H, ^2^H_2_ (10 mg/mL) were obtained using a Varian Inova-500 NMR spectrometer (Agilent Technologies, Santa Clara, CA) with VNMRJ 2.2D software. The chemical shifts were analyzed by MestReNova (https://mestrelab.com). The average DP of material is calculated using the following equation: DP = (H_1_ + H_pNP_)/H_pNP_. The signal H_1_ (≈4.30 ppm) corresponds to the anomeric protons of internal β-(1→4)-linked glucose units of cellulose, while H_pNP_ (≈5.3 ppm) is assigned to the anomeric proton of the p-nitrophenyl (pNP)-labeled reducing-end glucose unit.^37^

#### 2.4.3 X-ray Diffraction (XRD)

The insoluble cellulose-pNP and unlabeled cellulose were lyophilized for X-ray diffraction (XRD) measurements. The XRD was performed using an Anton Paar XRDynamic Powder diffractometer in Bragg–Brentano geometry. The diffraction was carried out using a background free sample holder (Si) in the 2θ range from 5° to 50° with a step size of 0.02°. The crystallinity index (CI) was measured based on the peak height measurements using the Segal method. The Segal Crystallinity index (CrI, %) was calculated using the relationship, $\mathrm{CrI}=\frac{\left(I_{002}-I_{\mathrm{am}}\right)}{I_{002}}100$ , wherein I_002_ is the maximum intensity of 020 peak at 21.7° 2θ, and I_am_ is intensity at 16° 2θ.

#### 2.4.4 Atomic Force Microscopy (AFM) Measurements

AFM measurements were performed using a commercial AFM system (Dimension FastScan Bio, Bruker, Billerica, MA) equipped with a Nanoscope V controller and operated via the associated software (Nanoscope 9.2, Bruker). All measurements were carried out in tapping mode under liquid conditions using FastScan D probes (Bruker) with nominal resonance frequency, spring constant, and tip radius of 110 kHz, 0.25 N/m, and 5 nm, respectively, as described previously.^20^ Samples for AFM imaging were prepared as follows: cellulose-pNP dispersed in water (∼4 mg/mL, 60 μL) was deposited onto freshly cleaved mica or highly oriented pyrolytic graphite (HOPG, grade I, SPI Supplies) substrates (each ∼1 cm^2^) and air-dried (16 h at room temperature). Prior to imaging, the substrate was rinsed with deionized water, and residual droplets were removed by gently spraying carbon dioxide on the surface. Before wetting, hydrophilic mica was surrounded by a hydrophobic boundary created by using commercially available nail polish. The substrate was then mounted onto the liquid stage of the AFM and wetted with 220 μL of buffer (50 mM sodium acetate buffer, pH 5.0). Reference images were recorded prior to enzyme addition, after which degradation was initiated by adding 60 μL *Tr*Cel7A (100 μg/mL). Image analysis was performed using an in-house MATLAB routine^38^ and *Gwyddion* 2.68.^39^

### 2.5 Thin-Layer Chromatography (TLC)

The supernatant of enzymatic reactions and controls was analyzed. A total of 30 μL of supernatant was concentrated (by heating) to 5 μL and spotted on a TLC instrument (Silica gel 60 F_254_ Merck, Darmstadt, Germany). Reference standard mixture was made by mixing the soluble pNP-oligosaccharides preparation (composition can be seen in Figure S1) with the commercially available unlabeled oligosaccharides (G1–G4 at ∼5 mM final concentration each). Refer to Table S1 for Rf values of different compounds present in the reference standard mixture. For the cellulose-pNP degradation reactions, the mobile phase comprised of 1-butanol, 1-propanol, and water (20:50:30, by volume) was used. Staining was done with the thymol reagent (thymol/ethanol/H_2_SO_4_, 0.5:95:5, w/v/v).

### 2.6 Protein Determination

The Roti-Quant or Roti-Nanoquant assay calibrated with BSA was used for protein determination. Ultraviolet (UV) absorbance measured on a Nanodrop 1000 spectrometer (Thermo Fisher Scientific, Waltham, MA) was used additionally with *Tr*Cel7A. Molar extinction coefficient calculated from the sequence (*Tr*Cel7A: 86750 M^–1^cm^–1^) was used. The concentrations of β-glucosidase (0.44 mg/mL) and *Tl*Cel7B (13.82 mg/mL) were used as declared by the commercial provider.

### 2.7 Enzymatic Degradation of Cellulose-pNP and Unlabeled Synthetic Cellulose

Reactions of cellulases and individual cellulase components were performed in 50 mM sodium acetate buffer, pH 5.0. Reactions of the cellulosome (native, disassembled) were performed in 30 mM sodium acetate buffer, pH 5.5, containing 100 mM NaCl, 10 mM CaCl_2_, 10 mM cysteine, and 2.0 mM EDTA. The “cellulosome buffer” was always prepared fresh on the same day of use.

Reactions were performed in a total volume of 1.0 mL in 1.5 mL Eppendorf tubes with an agitation rate of 600 rpm and a temperature of 50 °C (cellulases) or 55 °C (cellulosomes), using incubation in a Thermomixer comfort. Unless mentioned, the cellulose substrate concentration was 1.5 mg/mL and the enzyme concentration was 1.0 μg/mL. At certain times up to 4 h, a homogeneous sample (100 μL) was taken, heat inactivated (100 °C, 10 min), and centrifuged (12,400 *g*, 10 min, 4 °C, Eppendorf, 5418 R) to recover both the clear supernatant and the solid residue (Figure S2). The solid residue was used for MS analysis. The supernatant was supplemented with β-glucosidase (2.0 μg/mL) and incubated (50 °C, 500 rpm, 1 h). After heat treatment (∼100 °C, 10 min), the released pNP was determined in samples from the cellulose-pNP reaction. Samples were diluted 5-fold with 100 mM NaOH and pNP absorbance was measured at 405 nm (*e* = 18,500 M^–1^cm^–1^). The measured values were corrected for background (pNP reading from the cellulose-pNP incubation without enzyme; ≤ 20% of sample reading). In unlabeled cellulose reactions, glucose was measured (d-Glucose HK assay kit from Megazyme, Wicklow, Ireland). Enzymatic rates were determined from the initial part of the reaction time course (≤1 h) when the product release was approximately linearly dependent on time. Specific activities were calculated from these rates by normalizing on the protein concentration. One unit (U) of enzyme activity is the enzyme amount releasing 1 μmol pNP/min or 1 μmol chain length-averaged glucose/min. An average chain length of 7.4 was assumed for unlabeled synthetic cellulose.^18^

## 3 Results and Discussion

### 3.1 Synthesis of Cellulose-pNP

Purified CdP from *C. cellulosi* exhibited high activity with pNP-cellobioside (pNP-G2; 14.7 ± 0.1 U/mg; *n* = 3), comparable to cellobiose (12.2 U/mg) but markedly higher than that with pNP-glucoside (pNP-G1; 0.94 U/mg). Consequently, pNP-G2 was selected as the acceptor substrate despite its higher cost, ensuring efficient polymerization and minimizing side reactions. At 1 mL reaction scale, utilizing a 10:1 ratio of donor (100 mM α-glucose 1-phosphate) and pNP-G2 gave ∼50 mol % conversion of the donor, concomitant with formation of the solid material (30 mol % yield) (Figure 1A).

**Figure 1:**
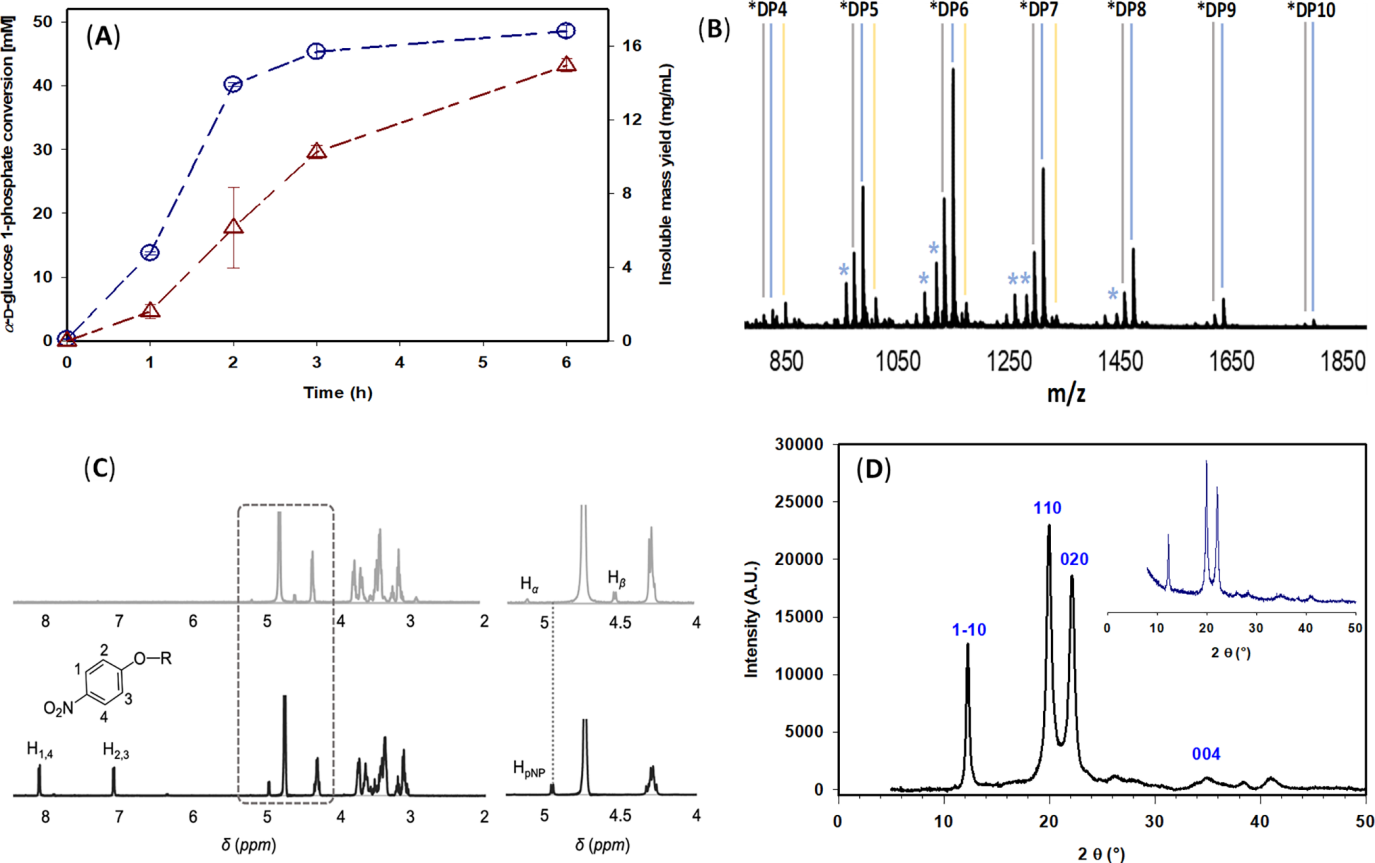
Synthesis and structural characterization of cellulose-pNP synthesized by CdP. (A) Time course of α-glucose 1-phosphate conversion (-Ο-), and insoluble mass yield (-Δ-). Reactions (*n* = 3) were performed with 100 mM α-glucose 1-phosphate, 10 mM pNP-G2, and 1.0 U/mL CdP. (B) MALDI-TOF MS spectra of cellulose-pNP after 6 h of synthesis. Gray and blue lines indicate Na^+^ and K^+^ adducts of cellulose-pNP, respectively. Yellow lines indicate mixed Na^+^ and K^+^adducts corresponding to [M + Na + K]. Blue stars indicate peaks corresponding to unassigned masses of fragmented ions. Refer to Figure S3 for detailed analysis of peak clusters observed for single oligosaccharide species in the cellulose-pNP. (C) ^1^H NMR spectra of cellulose-pNP (black) and unlabeled synthetic cellulose (gray) after 6 h of synthesis. The ^1^H NMR spectral region showing peaks for anomeric H_β_ and H_α_ in unlabeled cellulose and H_pNP_ in cellulose-pNP is enlarged in the right panel. (D) X-ray diffraction patterns of cellulose-pNP after 6 h of synthesis. The Miller indices of the main peaks are highlighted in blue. Diffraction patterns for unlabeled synthetic cellulose are shown in the inset.

Although optimization of CdP-catalyzed cellulose synthesis was not the objective of this study, strategies for improving yields have been reported previously.^29^ Considering the intended application of cellulose-pNP for enzymatic cleavage specificity studies, achieving a high anomeric purity was particularly important. The solid material was therefore characterized by MALDI-TOF MS and ^1^H NMR to confirm its composition and labeling fidelity. MALDI-TOF MS analysis (Figure 1B) showed that the material consisted of pNP-labeled oligosaccharides with degrees of polymerization (DP) between 4 and 10, centered at 6 (average DP ≈ 5.7). Each DP showed characteristic Na^+^ ([M + 23]) and K^+^ ([M + 39]) adducts as well as minor [M + 23 + 39] mixed adducts, where M represents the native oligosaccharide mass. Two additional peaks (M – 11 and M + 9), specific to the pNP-labeled material, indicated minor fragmentation of the parent pNP-oligosaccharides (Figure S3), consistent with earlier reports.^31^ Importantly, no signals corresponding to unlabeled cellulose were detected, confirming that CdP catalyzed the exclusive formation of the pNP-labeled material. Unlabeled cellulose contamination in CdP reactions with low-activity primers (e.g., 1-thio-β-glucose) arises from minor hydrolysis of α-glucose 1-phosphate, generating glucose as an alternative primer.^40^ Here, using the more reactive pNP-G2 (∼16-fold higher activity than pNP-G) effectively prevented this side reaction. ^1^H NMR spectra of cellulose-pNP (in 4% NaOD, D_2_O) showed characteristic aromatic signals of the 4-nitrophenyl group (7–8 ppm) without signals of α- and β-anomeric protons of the unlabeled reducing end (Figure 1C). This further underpinned the high reducing-end purity of the synthesized material.

Comparable enzymatic syntheses of cellulose-pNP have been achieved using glycosyltransferase (LgtB),^41^ glycosynthases (E414G variant of rice β-glucosidase),^42^ and *C. thermocellum* cellodextrin phosphorylases.^19,31^ The products obtained from glycosynthase and *C. thermocellum* CdP reactions exhibited similar distributions and average DP values to those in the present study. However, minor contamination was persistent in *C. thermocellum* CdP reactions, as reported by Pylkkänen et al.^31^ (their Figure S3), indicating incomplete labeling. Importantly, these materials were not evaluated for enzymatic cleavage specificity, which is a central focus of the current study.

### 3.2 Structural Characterization of Cellulose-pNP

X-ray diffraction analysis (Figure 1D) showed that cellulose-pNP exhibited high crystallinity (CI = 90) corresponding to cellulose allomorph II, consistent with the recent report.^31^ The crystallinity was comparable to Avicel (CI = 91.7) and bacterial microcrystalline cellulose (CI = 95.2).^43^ Unlabeled cellulose synthesized under identical conditions showed an analogous cellulose II structure (Figure 1D, inset). Cellulose allomorph II, characterized by an antiparallel chain arrangement, represents the thermodynamically most stable crystalline form of cellulose and is typical of regenerated cellulose.^12,29,44^

AFM imaging revealed that cellulose-pNP formed partially ordered, three-dimensional assemblies on hydrophilic mica surfaces (Figure 2). These appeared either as isolated, sheet-like structures typical of cello-oligosaccharide assemblies (Figure 2A) consistent with earlier reports^19,36,45^ and as larger aggregates composed of multiple sheets with distinct orientations (Figure 2B). Individual sheets exhibited a thickness of ∼4 nm (Figure 2C), which is comparable to the chain length of cellulose-pNP (DP ≈ 6) and are consistent with the recent report.^19^ AFM observations clearly suggest a molecular orientation perpendicular to the substrate, assuming a cellobiose length of roughly 1 nm per repeat unit.^46^ On hydrophobic HOPG, the assemblies appeared as circular features (∼3.5 nm in height, ∼200 nm in diameter; Figure 2D,E) that were more flexible, as indicated by incomplete tip tracking and multiple line artifacts.

**Figure 2:**
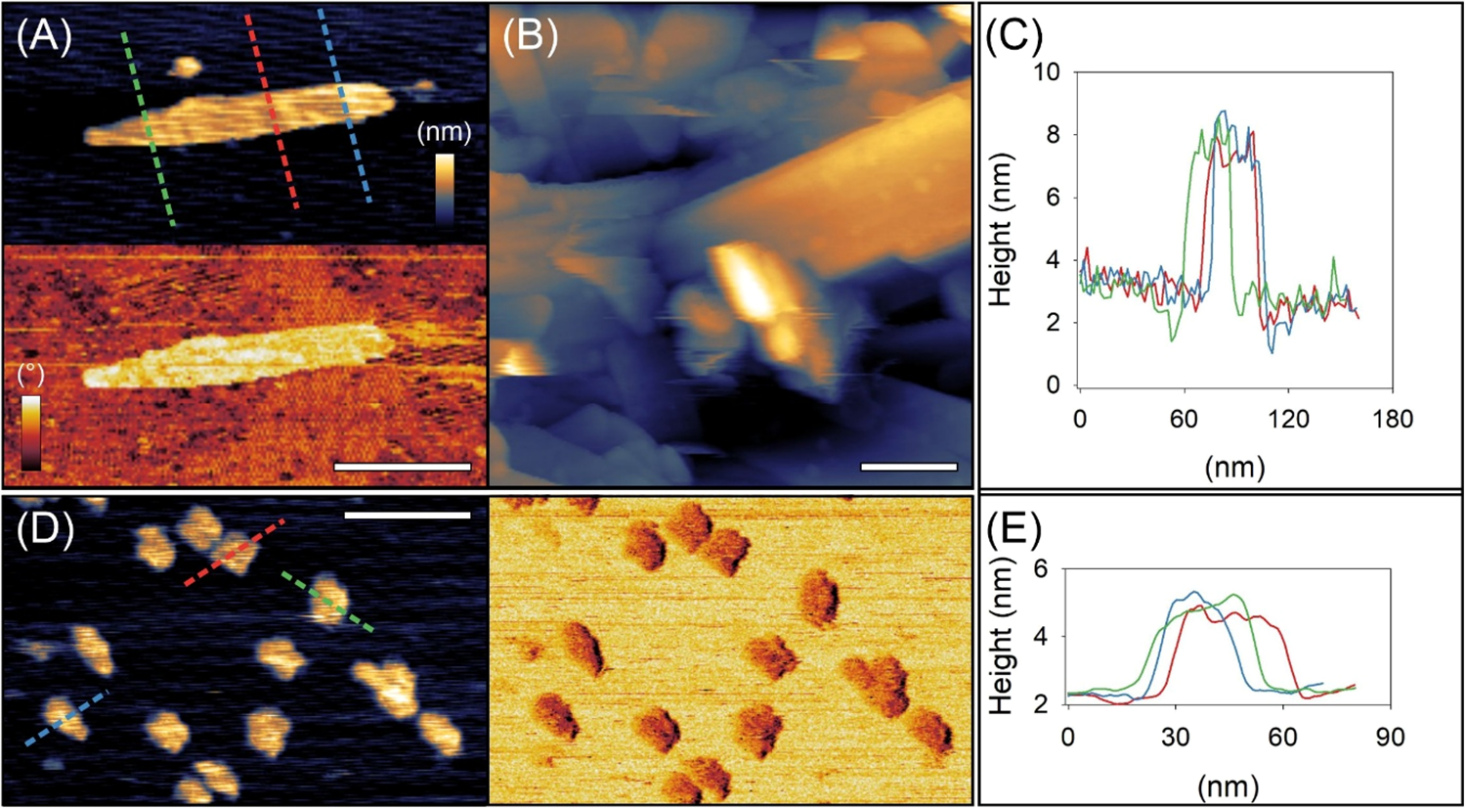
Structural characterization of cellulose-pNP assemblies in liquid. (A) Cellulose-pNP appears as isolated, sheet-like structures or (B) as larger aggregates composed of multiple overlapping sheets on mica. Panel A shows a height image (top) and the corresponding material-sensitive phase image (bottom). (C) Multiple height profiles recorded across an individual cellulose-pNP sheet shown in panel A; line color indicates the position of each profile. (D) Morphology of cellulose-pNP deposited on HOPG shown as height (left) and phase (right) images. (E) Height profiles of individual cellulose-pNP aggregates corresponding to panel D; line color indicates the profile position. Scale bars: 100 nm. False-color scales are shown in panel A. Height and phase ranges were 7 nm/54° (A), 140 nm (B) and 3.5 nm/29° (D), respectively.

Collectively, these morphological differences reflect distinct molecular organization on the two supports. On mica, cellulose-pNP formed rigid, crystalline-like sheets, whereas on HOPG, the assemblies were less ordered and exhibited more heterogeneous arrangements. While the observed differences could, in principle, arise from subtle substrate-induced effects, another explanation is that multiple supramolecular architectures coexist in solution prior to deposition, with surfaces selectively adsorbing different populations. Recent studies^45,47,48^ on synthetic cello-oligosaccharides and related cellulose derivatives demonstrate that small changes in chain-end chemistry or solution conditions can produce heterogeneous nanoscale assemblies, which display preferential adsorption depending on surface chemistry. In this view, mica may favor adsorption of larger, more rigid sheets, whereas HOPG retains more flexible or heterogeneous aggregates. Regardless of whether these differences originate primarily from solution-phase preassembly or surface-mediated organization during adsorption, the AFM data clearly show that cellulose-pNP forms mechanically and morphologically distinct assemblies on the two supports.

Supporting this interpretation, tapping-mode AFM phase images revealed reduced phase angles over HOPG assemblies, consistent with increased flexibility or lower packing density of the cellulose chains, whereas mica-bound sheets exhibited higher phase values indicative of more rigid or ordered regions. Although other factors such as tip–sample adhesion or local viscoelasticity could also contribute, the phase data align with the morphological trends observed in the height images.^49,50^

### 3.3 Enzyme Activities on Cellulose-pNP

The experimental workflow for analyzing the enzymatic cleavage of cellulose-pNP is shown in Figure S2. Because the soluble background can complicate interpretation of enzyme activity, we first assessed the physical stability of cellulose-pNP in aqueous suspension. A completely soluble preparation of pNP-labeled oligosaccharides of DP ≤ 5 (Figure S1, see section 2.3) was used as analytical reference. When cellulose-pNP was incubated without enzyme at 1.5 mg dry mass/mL (equivalent to 1.3 mM with the assumption of DP 6 cellulose chains), only ≤ 5% of the material was released as soluble oligosaccharides and remained constant over time (Figure S4). Enzyme adsorption to cellulose-pNP was confirmed in control reactions at a higher ratio (1:150), where ≥ 55% of added protein was bound to the substrate (Figure S5). Taken together, the physical stability of cellulose-pNP and the substantial enzyme adsorption emphasizes that the enzymatic turnover relies primarily on insoluble cellulose-pNP.

After validating the experimental conditions, we next analyzed a representative set of cellulase enzymes (Table S2) for hydrolysis of cellulose-pNP. To critically benchmark this substrate, unlabeled cellulose and Avicel were included as established insoluble references, enabling a direct assessment of how cellulose-pNP compares with conventional substrates. To ensure complete adsorption during activity measurements, we used enzyme-to-substrate ratios that were up to 10-fold more substrate-rich than the adsorption ratio identified in the 1:150 control experiments. Complete reaction time courses were recorded (Figures S6–S8) and rates were determined from the initial phase (Figure S6). Summarized data in Table 1 reveal that among individual cellulases, the internally chain-cleaving endocellulase (*Tl*Cel7B) was up to 3.7-fold more active than the chain end-cleaving cellobiohydrolases (*Tr*Cel7A and *Tr*Cel6A).

**Table 1:** Specific Activities of Enzymes on Cellulose-pNP in Comparison to Unlabeled Synthetic Cellulose and Avicel

	specific activity (U/mg)		
enzymes	cellulose-pNP	unlabeled cellulose^a^	Avicel^b^
*Tl*Cel7B	6.1 ± 0.2	6.8 ± 1.2	0.035 ± 0.006
*Tr*Cel7A	1.6 ± 0.1	3.9 ± 0.3	0.048 ± 0.003
*Tr*Cel6A	2.8 ± 0.1	7.9 ± 2.1	0.056 ± 0.01
cellulases	4.3 ± 0.2	8.1 ± 0.02	0.52 ± 0.02
cellulosome	1.5 ± 0.1	2.0 ± 0.07	0.102 ± 0.001
disassembled cellulosome	0.7 ± 0.03	1.8 ± 0.2	0.03 ± 0.003

a Activities were normalized as per avg. DP (∼7.4) of unlabeled cellulose to compare with the activities obtained using the cellulose-pNP.

b Individual cellulases were used at 10.0 μg/mL. The cellulase mixture, the cellulosome and the disassembled cellulosome were used at 5.0 μg/mL.

Among the cellobiohydrolases, the enzyme cleaving from the non-reducing end (*Tr*Cel6A) was more active than *Tr*Cel7A that cleaves from the opposite chain end.^12^ Comparison of activities with cellulose-pNP and unlabeled cellulose (Table 1) showed the *Tl*Cel7B to have been unaffected by the pNP reporter group whereas the cellobiohydrolases were ∼2.3-fold less active with cellulose-pNP. The reducing-end pNP group has a direct effect on the chain-end cleavage by *Tr*Cel7A. Chain cleavage from the non-reducing end by *Tr*Cel6A is arguably affected by the pNP groups of neighboring cellulose chains in antiparallel orientation to the chain under attack.

Comparison with unlabeled cellulose affirms the reporter group in cellulose-pNP to have only a small effect on enzyme specific activity (≤2-fold decrease). The study of individual cellulases shows the cellobiohydrolases to be affected in their activity (≤2.8-fold) by the pNP group, whereas the endoglucanase was barely affected. Haataja and co-workers^51^ show that the kinetics of family GH7 cellobiohydrolases (i.e., *Tr*Cel7A) on soluble pNP-G2 is critically affected by nonproductive binding. However, pNP-G2 is a poor substrate of *Tr*Cel7A^51^ and we hardly saw its degradation once the pNP-G2 was released from cellulose-pNP by the *Tr*Cel7A (see later in section 3.4). Generally, the specific activities of the cellulases on cellulose-pNP (Table 1) were comparable, roughly within the same order of magnitude, with the specific activities of these enzymes on soluble cello-oligosaccharides of suitable DP (endoglucanases;^16^ cellobiohydrolase I;^52^ cellobiohydrolase II^52^). The synthetic cellulose was much more reactive (≥100-fold) with individual cellulases than Avicel. Specific enzyme activities on cellulose-pNP are considerably higher (≥8-fold) than on Avicel (Table 1).

The native mixture of cellulases was about as active as the most active of the individual enzymes, *Tl*Cel7B on cellulose-pNP and *Tr*Cel6A on unlabeled cellulose (Table 1). The result contrasts to the enzyme activities on microcrystalline cellulose: the cellulase mixture was ∼10-times more active on Avicel than the individual enzymes. Enzyme synergy in hydrolysis may be absent on the synthetic cellulose, or at least it was considerably lower than that on Avicel.

We also analyzed the *C. thermocellum* cellulosome in two forms: (1) the intact complex, comprising typically nine enzymatic subunits assembled on the scaffoldin and (2) the disassembled “cellulase-like” mixture obtained after releasing the enzymatic subunits from the scaffoldin.^20^ The intact cellulosome was less active (≥ 3-fold) than the cellulases on all cellulose preparations used (Table 1). The disassembled cellulosome exhibited lower activity than the intact cellulosome on cellulose-pNP and Avicel, except on unlabeled synthetic cellulose, which was hydrolyzed by the two cellulosome preparations with equal activity. Together, these results establish cellulose-pNP as a practical and reactive surrogate for insoluble cellulose, providing a generally useful model substrate for assaying the activity of cellulolytic enzymes.

### 3.4 Mapping the Hydrolytic Cleavages in Cellulose-pNP

Because mapping chain-cleavage patterns requires positional information provided uniquely by the pNP label, cleavage specificity was analyzed only with cellulose-pNP using end-specific processive cellulases and multicellulase systems. Determination of enzymatic chain cleavage patterns in cellulose-pNP (Figures 3 and 4) confirms, but also complements, earlier works done with soluble, labeled, or unlabeled cello-oligosaccharides.^52–54^ In particular, MS analysis of the solid residue from the enzymatic reaction provides important additional information. Characterization of the individual cellobiohydrolases is summarized in Figure 3. The product pattern from the *Tr*Cel7A reaction is consistent with processive chain cleavage from the reducing end.^13^ The soluble products found in TLC were pNP-G, cellobiose (G2, main), and cellotriose (G3, minor). The insoluble residue was largely devoid of pNP label (88%), the main DP was 4 (Figure 3A). To estimate the primary cleavage action of *Tr*Cel7A on cellulose-pNP, TLC of soluble products (after 4 h of incubation) was visualized under UV light at 254 nm (Figure S9), selectively detecting pNP-containing species. Semiquantitative densitometric analysis using ImageJ indicated that the pNP-labeled soluble products consisted of pNP (24%), pNP-G1 (59%), and pNP-G2 (17%), clearly demonstrating that the primary initial cleavage occurs after the pNP-G1 unit. Our results are in excellent agreement with reported cleavage-frequency measurements of *Tr*Cel7A on [1-^3^H]-labeled G4–G6 oligosaccharides, where cleavage at the second glycosidic bond from the reducing end was observed with a frequency of 0.4–0.7.^55^ The product pattern of the *Tr*Cel6A reaction indicates chain cleavage from the non-reducing end. The soluble products were G2 and pNP-G3 (main) as well as G3 and pNP-G2 (minor) (Figure 3B). The solid residue mostly comprised pNP-labeled products of DP considerably larger than in the reaction of *Tr*Cel7A. The result suggests that part of the pNP-oligosaccharides assembled into the cellulose-pNP material are not well accessible to *Tr*Cel6A as it interacts with the solid substrate.

**Figure 3:**
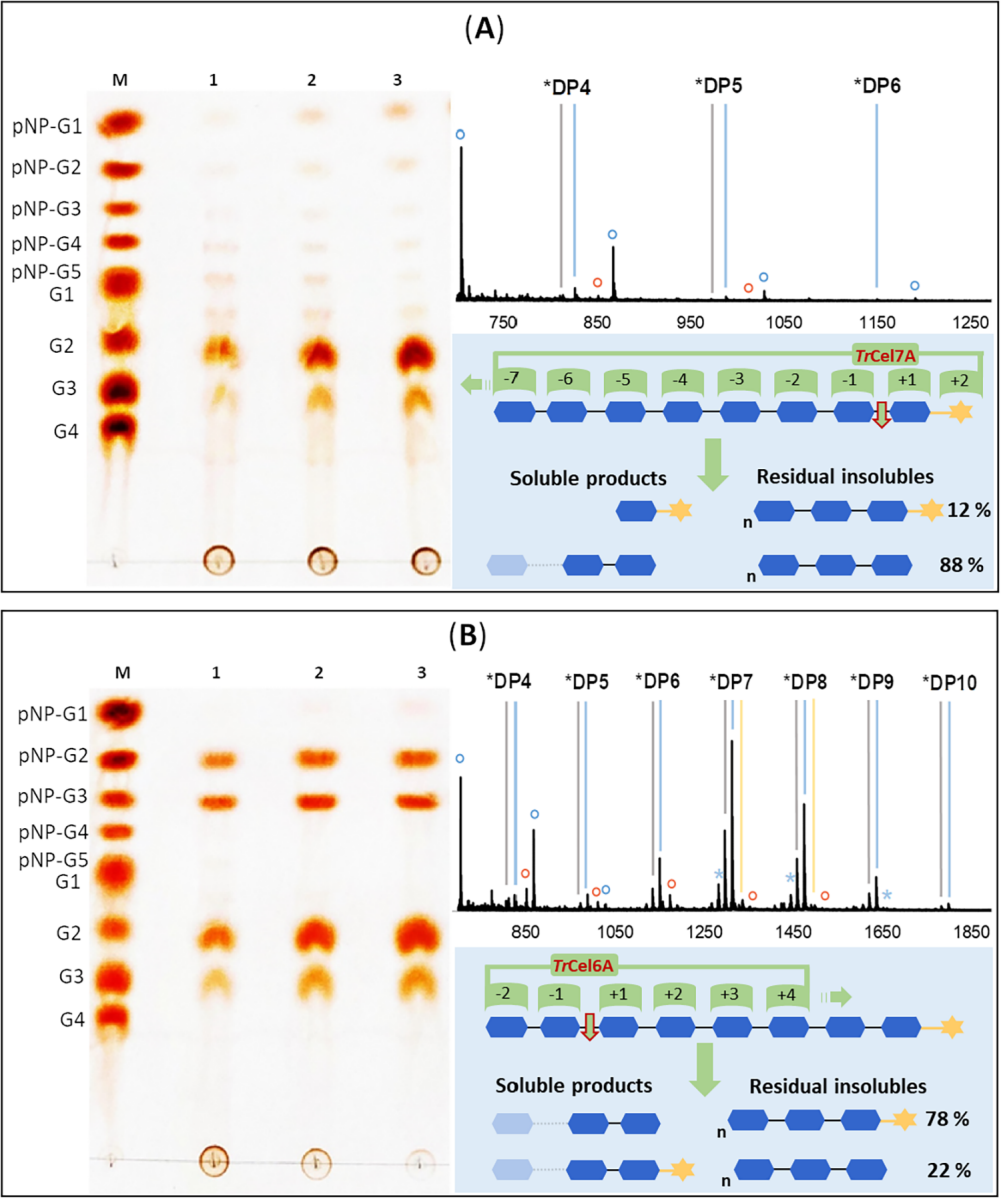
Analysis of the chain cleavage specificity of (A) *Tr*Cel7A and (B) *Tr*Cel6A acting on cellulose-pNP. For each panel, the left image shows the TLC analysis with an oligosaccharide standard mixture (lane M) and supernatants collected after 0.5, 1.5, and 4 h of reaction (lanes 1–3). The upper right image shows the relevant region of the MALDI-TOF MS spectra of the solid residue after 0.5 h. pNP-labeled oligosaccharides are shown as lines (gray for Na^+^ adducts and blue for K^+^ adducts), whereas unlabeled oligosaccharides are shown as circles (red for Na^+^ adducts and blue for K^+^ adducts). The lower right image depicts the proposed cleavage mode, based on the reported subsite binding of oligosaccharides in the two enzymes.^13^ Semitransparent symbols indicate additional dominant products (e.g., G3 in *Tr*Cel7A), schematically represented to optimize figure space. Connected oligosaccharide symbols with superscript n denotes multiple repeating units. The percent composition of the solid residue is based on MS measurements.

**Figure 4:**
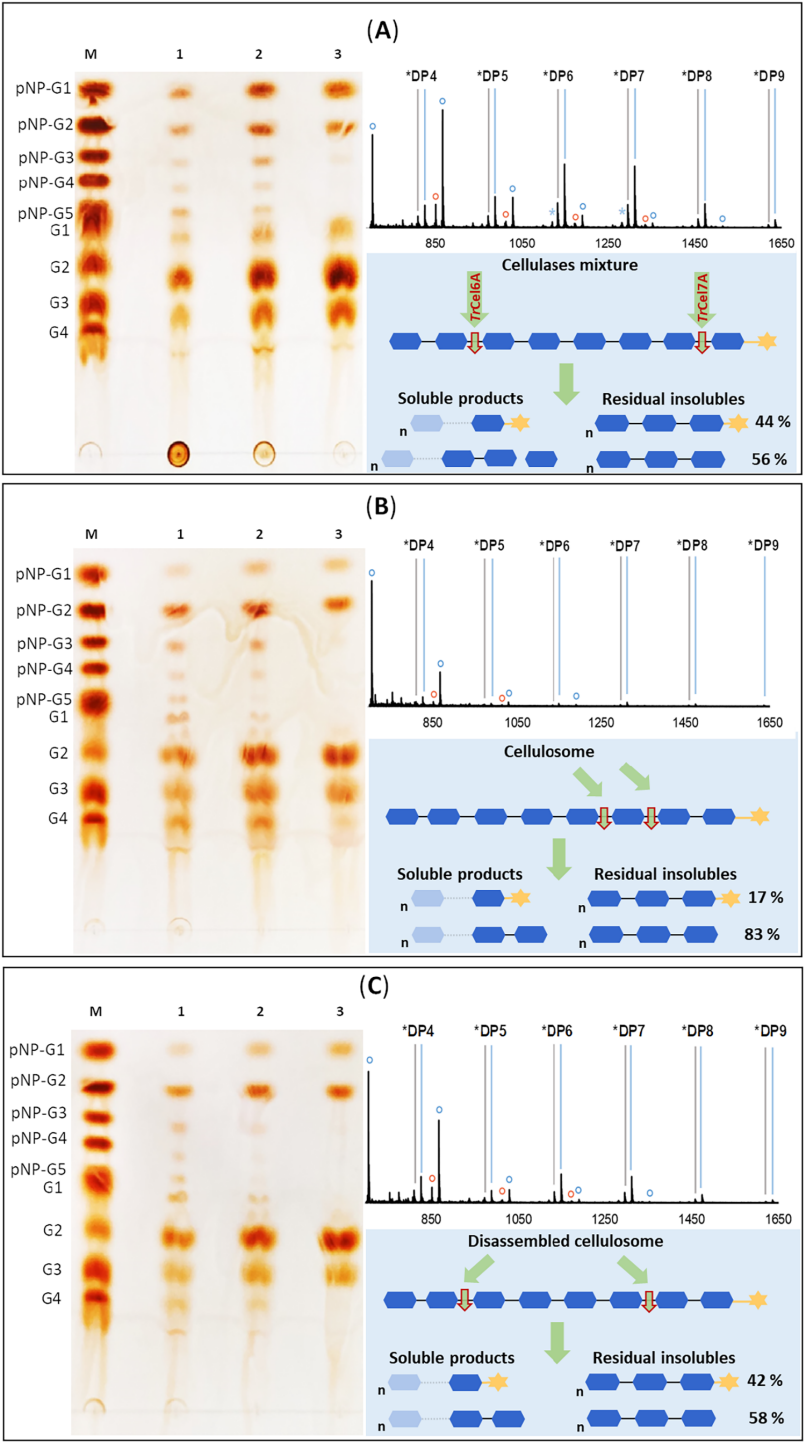
Analysis of the chain cleavage specificity of (A) the *T. reesei* cellulases mixture, (B) the *C. thermocellum* cellulosome, and (C) the disassembled cellulosome acting on cellulose-pNP. The panel layout and symbol conventions in the MALDI-TOF MS spectra are as described in Figure 3. The lower right image depicts the proposed cleavage modes in different enzyme systems. Semitransparent symbols indicate additional dominant products, schematically represented to optimize figure space. Connected oligosaccharide symbols with superscript n denotes multiple repeating units. The percent composition of the solid residue is based on MS measurements.

Chain cleavages by the native ensemble of cellulases resulted in a solid residue with about half of the chains devoid of the pNP label (Figure 4A). The unlabeled chains were shorter (DP 4 main, DP 5 minor) than the pNP-labeled chains (DP 6–7 main; DP 8–9 minor). The soluble products were G2 (main), G3 and glucose (minor). pNP-G2 and pNP-G were also released. The cellulase mixture contains a low level of β-glucosidase activity (0.4 U/mg; pNP-G) which arguably explains the minor formation of glucose (Figure 4A) that was not observed in reactions of the individual enzymes (Figure 3). The products of cellulase activity seemed to reflect mainly the chain cleavages by the two cellobiohydrolases, promoting chain degradation from both ends. Chain-internal cleavages are ruled out as the predominant form of chain degradation because initial cleavage products (e.g., labeled and unlabeled species of DP ≥ 3) were formed in a minor amount.

The product pattern of cellulose-pNP hydrolysis by the intact cellulosome (Figure 4B) was different from that of the cellulases (Figure 4A). The solid residue involved unlabeled oligosaccharide chains to a large extent (83%) and the main DP was 4. The soluble products comprised G2 and pNP-G2 mainly. While pNP-G was prominent in the cellulase reaction, it was missing from the cellulosome reaction (Figure 4B). The cellulosome thus attacks the cellulose-pNP primarily from the reducing end. Disassembly of the cellulosome caused a change in the pattern of products released toward greater similarity with the cellulase reaction (Figure 4C). This was noted especially in the solid residue that contained portion of pNP-labeled chains substantially larger than in the reaction of intact cellulosome, yet approaching the balanced distribution of labeled and unlabeled chains as in the cellulase reaction.

### 3.5 AFM Analysis of Enzymatic Degradation of Cellulose-pNP

AFM degradation studies were performed exclusively with the processive cellulase *Tr*Cel7A, as this enzyme is not only most abundant in cellulases mixture but also mechanistically best-understood model exocellulase and provides the clearest interpretation of structure–activity relationships.^12,13^ Enzymatic degradation were performed specifically on mica, as the low image quality on HOPG prevented reliable analysis. Upon enzymatic treatment, larger cellulose aggregates exhibited characteristic morphological changes: individual sheets appeared mobile and were occasionally displaced by the AFM tip during imaging (Movie S1). Degradation proceeded preferentially from the sheet edges, rather than from the center (Figure 5C,E), leading to a gradual reduction of the overall area (Movie S1 and Movie S2). In several cases, directed degradation along a specific axis was observed (Figure 5A,B). Another example is shown in Figure 5C,D, where at least one sheet was partially removed in a directed fashion (red rectangle) without visibly affecting an overlying sheet. This likely represents the degradation of a double-sheet structure (∼10 nm in height, Figure 5D) or an aggregate composed of higher-DP chains. Isolated sheets on mica remained largely unaffected during enzymatic exposure (Movie S3), even though individual enzyme particles could be identified along their edges (Figure 5E).

**Figure 5:**
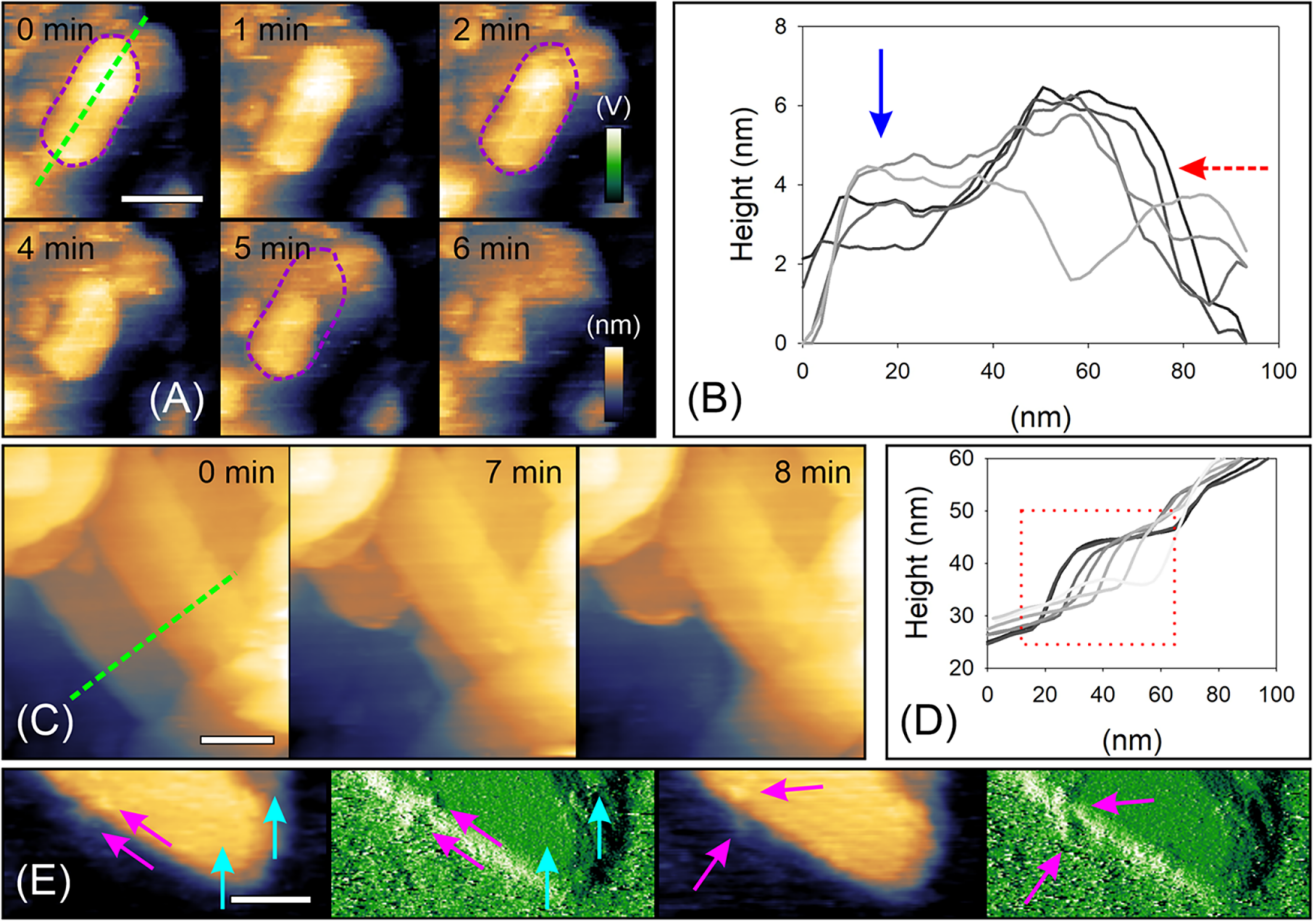
*Tr*Cel7A activity on cellulose-pNP assemblies. (A) Time-lapse AFM images showing predominantly directional degradation of a single cellulose sheet exposed within a cellulose-pNP sheet assembly. The initial contour of the sheet is outlined in purple. (B) Height profiles recorded along the green line in panel A. Time points are represented by the line color, ranging from black to light gray (greyscale). The red arrow indicates the main directions of degradation, while the blue arrow marks a region that remained largely unaffected. Note that due to the degradable nature of the support, height fluctuations may exceed typical values. (C) Exemplary time-lapse images of another cellulose-pNP structure and (D) corresponding height profiles recorded along the green line in panel C. Time is expressed by the line color (greyscale), and the red rectangle highlights the region of active degradation. (E) Representative height and amplitude images showing *Tr*Cel7A molecules (magenta arrows) located at or near the edge of approximately two stacked cellulose sheets, indicated by the height (∼10 nm) and amplitude channel (cyan arrows mark the two plateaus). Scale bars: 50 nm. False-color scales are shown in panel A. Height and amplitude ranges were 15 nm (A), 70 nm (C), and 15 nm/17.5 V (E), respectively.

The directed degradation observed in some cases suggests that enzyme activity can be locally guided, potentially by chain orientation or local structural context. This is consistent with the mechanistic understanding that exocellulases processively cleave chains from accessible ends,^13^ and that the local organization of multiple chains into an aggregate can influence enzyme binding and processivity.^11^ The reduced activity on isolated or double sheets implies that the surrounding microenvironment within larger aggregates facilitate hydrolysis. We therefore propose that cooperative interactions with adjacent sheets or additional carbohydrate-binding opportunities provided by slightly misaligned neighboring layers may help in stabilizing the enzyme and promote productive cleavage. Based on these considerations, we suggest a conceptual model in which *Tr*Cel7A binding and activity on solid cellulose substrates is not solely determined by chain orientation, but is strongly modulated by local substrate architecture (Figure 6).

**Figure 6:**
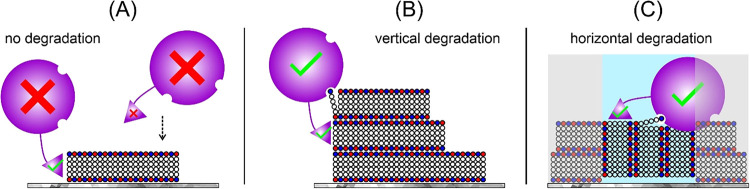
Schematic representation of *Tr*Cel7A interactions with cellulose-pNP under distinct structural arrangements. *Tr*Cel7A is shown with its CBM (triangle) and catalytic core (sphere). Cellulose-pNP chains are represented as glucose units (open circles), with non-reducing ends (red) and reducing ends (blue). (A) Antiparallel single-chain configuration on a support restricts productive binding, allowing only CBM engagement while preventing catalytic core access. (B) Stacked, perpendicularly oriented chains permit CBM binding at a chain terminus and enable catalytic engagement with an adjacent sidewall chain, supporting vertical degradation. (C) Mixed perpendicular and vertically oriented aggregates allow pseudo-processive action of *Tr*Cel7A on vertically aligned chains, while perpendicular sheets (gray) remain inaccessible.

On single cellulose sheets immobilized on a support (Figure 6A), *Tr*Cel7A would be expected to have limited access to the top surface due to steric hindrance. The CBM (triangle) may productively bind to the sidewall; however, in this configuration, the catalytic core (sphere) may remain out of contact with the surface owing to geometric constraints. In contrast, when multiple cellulose sheets are stacked (Figure 6B), the CBM could attach to an adjacent sheet, enabling the catalytic core to engage a chain along the sidewall of a neighboring sheet. In mixed perpendicular and vertically oriented sheet arrangements (Figure 6C, a), a subset of chains (highlighted in light blue) may be oriented in a way that makes them accessible to *Tr*Cel7A, allowing pseudo-processive activity, whereas other sheets (gray background) remain inaccessible.

While the configurations depicted in Figure 6 are derived from surface-bound assemblies imaged by AFM, we cannot exclude the possibility that a fraction of the hydrolyzable cellulose-pNP material may already form partially associated or multilayered structures in aqueous dispersion prior to deposition. Such pre-assembled supramolecular organization has been reported for short cellulose oligomers and could contribute to the enhanced degradation observed for multi-sheet aggregates.^45,48^ At the same time, the geometric context at a solid–liquid interface differs from that in free solution. Surface association can influence sheet orientation and the fraction of chains exposed to the enzyme, whereas assemblies dispersed in solution would be expected to present more uniformly accessible surfaces without support-induced asymmetry. This distinction is consistent with the well-established ability of *Tr*Cel7A to hydrolyze soluble cello-oligosaccharides^55,56^ and emphasizes that substrate architecture and local environment jointly influence enzymatic accessibility.

Collectively, these results suggest that multi-sheet aggregates provide a structural orientation that supports processive enzymatic degradation, whereas isolated sheets appear less accessible and more resistant. These observations highlight the value of high-resolution AFM imaging for revealing how nanoscale morphology and local mechanical properties of insoluble cellulose influence enzymatic activity. Although the precise molecular arrangements cannot be directly resolved under the present imaging conditions, AFM provides insights that are difficult to obtain from studies in a solid suspension alone.

## 4 Conclusions

Synthetic cellulose-pNP was obtained from the chain oligomerization reaction of *C. cellulosi* CdP with pNP-G2 as the acceptor. The cellulose chains exhibit an average degree of polymerization of ∼5.7 and self-assemble into highly crystalline (CI 90%) solid material of allomorph II crystal structure. The synthetic material shows high purity (≥ 95%) regarding the pNP label present at the reducing end of the cellulose chains. AFM imaging further revealed that cellulose-pNP self-assembles into partially ordered, sheet-like nanostructures on hydrophilic surfaces, while forming more disordered and flexible aggregates on hydrophobic supports. The ordered sheets exhibit nanoscale heights (4 nm) consistent with the expected degree of polymerization and display higher phase contrast, indicating greater stiffness and crystalline order. In contrast, the amorphous aggregates on HOPG show locally reduced phase angles, consistent with lower packing density and increased energy dissipation. The cellulose-pNP was used to characterize the activities and chain cleavage specificities of cellulose-degrading enzymes, with the possibility to analyze soluble and insoluble products of enzymatic chain cleavage. The results reaffirm the cleavage modes of cellulases previously characterized with soluble reducing end-labeled oligosaccharides. They provide new insight into the oligosaccharide cleavage mode of the cellulosome and show the role of assembly of the enzymatic subunits of the cellulosome for overall cleavage specificity. AFM imaging of enzymatic hydrolysis shows that *Tr*Cel7A preferentially attacked the edges of the cellulose-pNP sheets, resulting in gradual erosion of the overall area. Degradation often proceeded along specific directions and was markedly reduced or absent on isolated or small sheet regions, suggesting that the local environment and supramolecular packing within aggregates facilitates degradability. Overall, our study demonstrates the versatile utility of cellulose-pNP in the study of enzymes catalyzing cellulose depolymerization.

## Supplementary Material









## Data Availability

All data are included in the manuscript. Materials are available from the corresponding author (B.N.) upon reasonable request.

## Notes

TLC analysis of the soluble pNP-oligosaccharides; schematics of the assay development using cellulose-pNP; closeup of MALDI-TOF MS spectra of cellulose-pNP and unlabeled cellulose; TLC for the physical stability of cellulose-pNP; *T. reesei* cellulases adsorption on cellulose-pNP; time courses of enzymatic hydrolysis of cellulose-pNP; Avicel and unlabeled synthetic cellulose; TLC analysis to estimate the primary cleavage action of *Tr*Cel7A; Rf values of different compounds present in the reference standard mixture; and description of the set of enzymes used in the current study (PDF)
Time-lapse observation of *Tr*Cel7A acting on cellulose-pNP aggregates (AVI)
Snapshots showing partially directional degradation by *Tr*Cel7A (AVI)
Isolated sheets on mica shown to remain largely unaffected during enzymatic exposure (AVI)
