# Supplementary material for: Reporter Group-Labeled Synthetic Cellulose: Structural Characterization and Utilization in Mapping the Cellulose Chain-Cleavage Modes of Cellulases
Source: Biomacromolecules. 2026 Feb 25;27(3):2163–74. doi: 10.1021/acs.biomac.5c02515 (PMC12977043; doi:10.1021/acs.biomac.5c02515)
Supplement: Supplementary file 1 [file bm5c02515_si_001.pdf]

Supporting Information for:

Reporter group-labeled synthetic cellulose: Structural characterization and utilization in mapping the cellulose chain cleavage modes of cellulases

Gaurav Singh Kaira[1,2], Manuel Eibinger[1], Chao Zhong[1], and Bernd Nidetzky[1,2,*]

[1]Institute of Biotechnology and Biochemical Engineering, Graz University of Technology, NAWI Graz, Graz, Austria

[2]Austrian Centre of Industrial Biotechnology (acib), Graz, Austria

*Corresponding author: Bernd Nidetzky; bernd.nidetzky@tugraz.at

Authors: Gaurav Singh Kaira; gaurav.kaira@tugraz.at, Manuel Eibinger; m.eibinger@tugraz.at, Chao Zhong; czhong@tugraz.at

Table of Contents **Page No.**

1. TLC analysis of the soluble pNP-oligosaccharides preparation S-3
2. Schematics of enzyme assay using cellulose-pNP S-4
3. Closeup of MALDI-TOF MS spectra of cellulose-pNP and unlabeled cellulose S-5
4. Analysis of the physical stability of cellulose-pNP in buffer S-6
5. *T. reesei* cellulases adsorption on cellulose-pNP S-7
6. Time courses of enzymatic hydrolysis S8-S10
7. TLC analysis to estimate the primary cleavage action of *Tr*Cel7A S11
8. Tables for Rf values of TLC standard and description of different enzymes used S12-S13
9. Supporting movie captions S-14

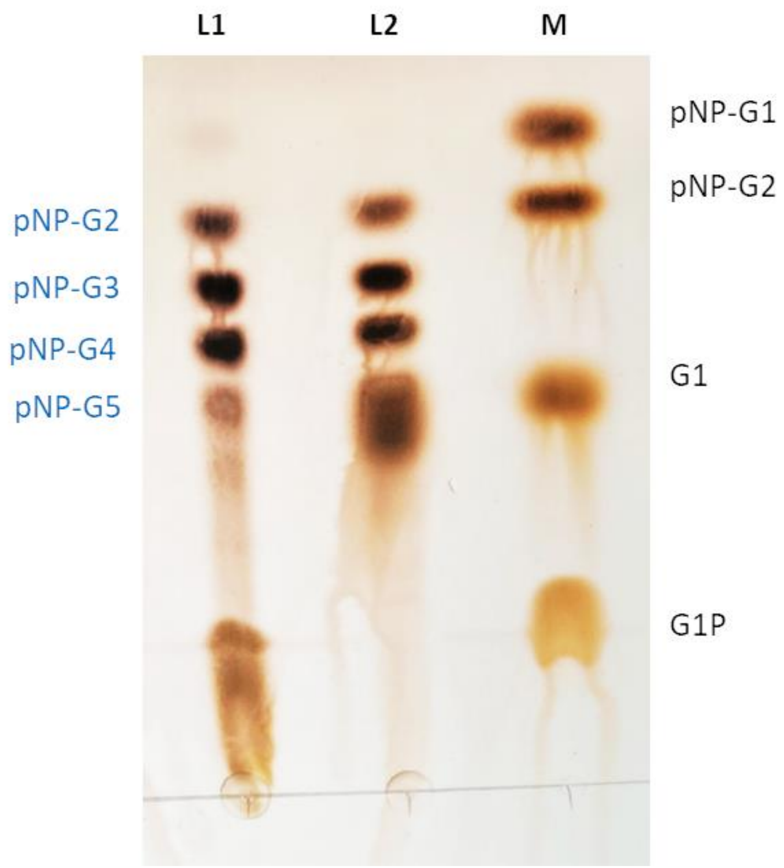

Figure S1. TLC analysis of the soluble pNP-oligosaccharides preparation. Lane 1 indicates soluble pNP-oligosaccharides mixture synthesized by CdP. Lane 2 indicates the soluble mixture after phosphatase treatment to remove the residual α-glucose 1-phosphate. Lane M indicates standard mixture. Expected pNP-labeled oligosaccharides in the soluble preparation are highlighted in blue.

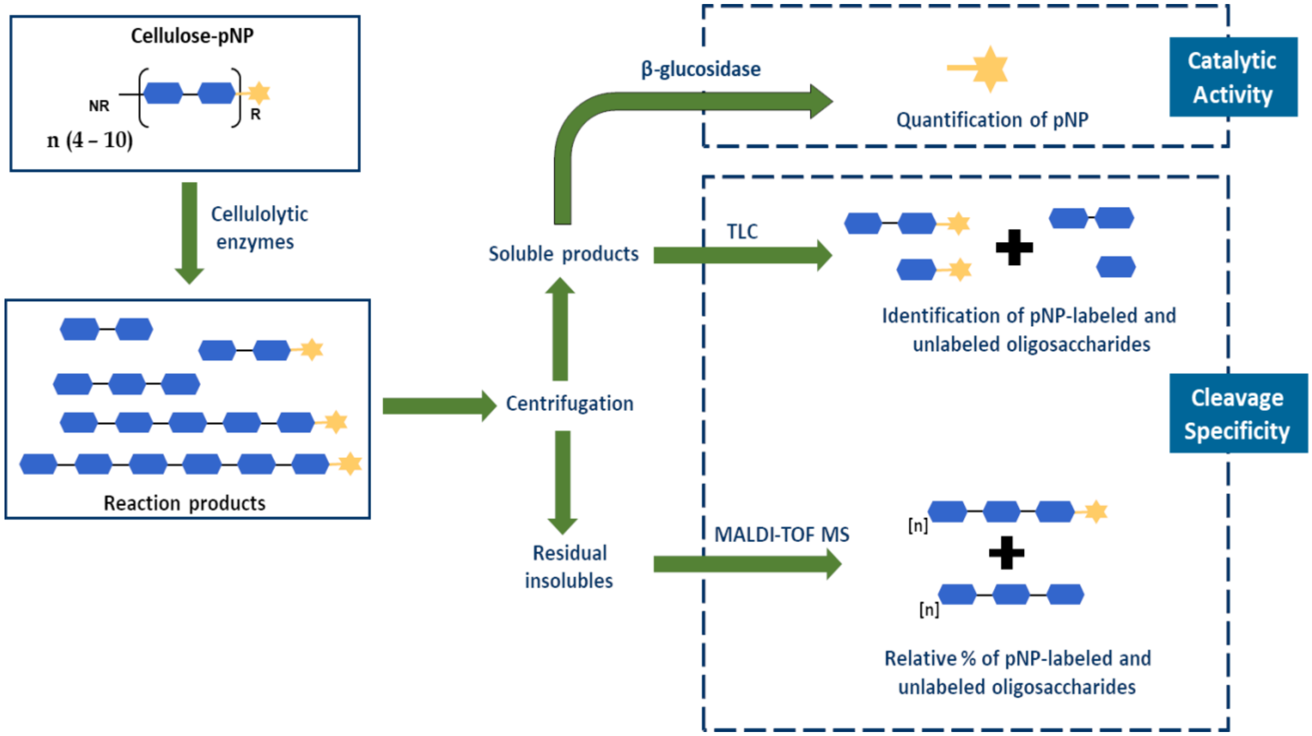

Figure S2. Schematics of the experimental workflow for analyzing the catalytic activity and cleavage specificity of different enzymes using cellulose-pNP. A set of enzymes (listed in Table S1) was employed to cleave the cellulose-pNP. Enzyme catalyzed reaction products were separated to soluble and insoluble products by centrifugation. Soluble products were hydrolyzed by β-glucosidase to quantify 4-nitrophenol (by absorbance 405 nm). Enzymatic activity was determined based on the rate of pNP release from the linear phase of the cellulose-pNP degradation time course. Additionally, the soluble products were analyzed by TLC to characterize activity profiles. The residual insoluble material was analyzed by MALDI-TOF MS, where the relative percent population of pNP-labeled and unlabeled material were calculated. The TLC and MALDI-TOF MS results were collectively assessed to map the cleavage specificity of different enzymes.

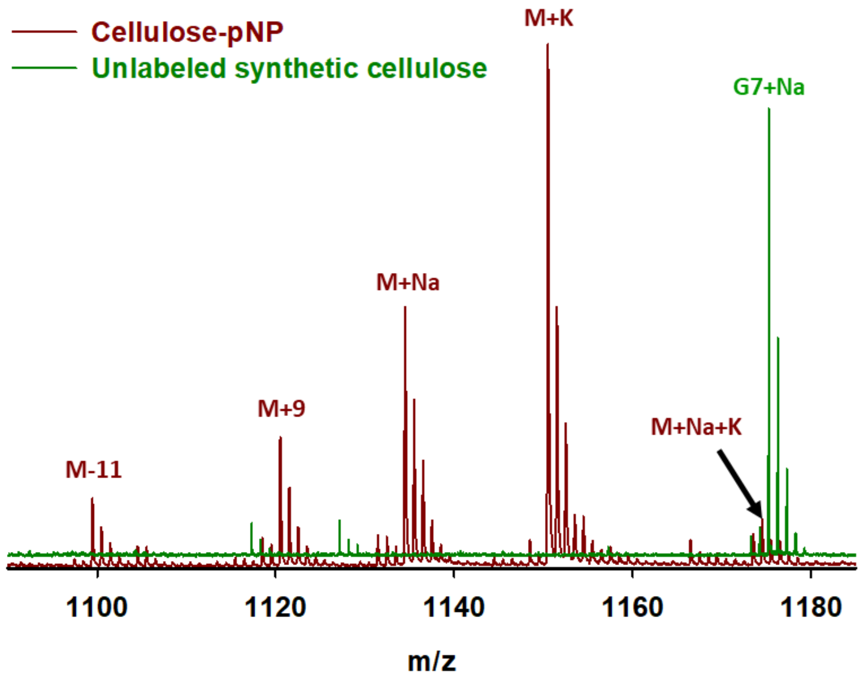

Figure S3. Close up view of MALDI-TOF MS spectra displaying typical clustering of peaks observed for cellulose-pNP (red). The most abundant pNP-labeled oligosaccharide (pNP-G6) of cellulose-pNP is focused here. For each mass of pNP-labeled oligosaccharide (M), total of five peak clusters are evident. Three of these clusters correspond to adducts with Na^+ [M + 23], K^+ [M + 39] and a combination of Na^+ and K^+ [M + 23 + 39]. The remaining two clusters (M – 11) and (M + 9), suggest fragmentation of parent pNP-oligosaccharide. Notably, these unidentified peaks (M - 11; M + 9) appears to be specific to cellulose-pNP, as they are essentially absent in the unlabeled synthetic cellulose (green).

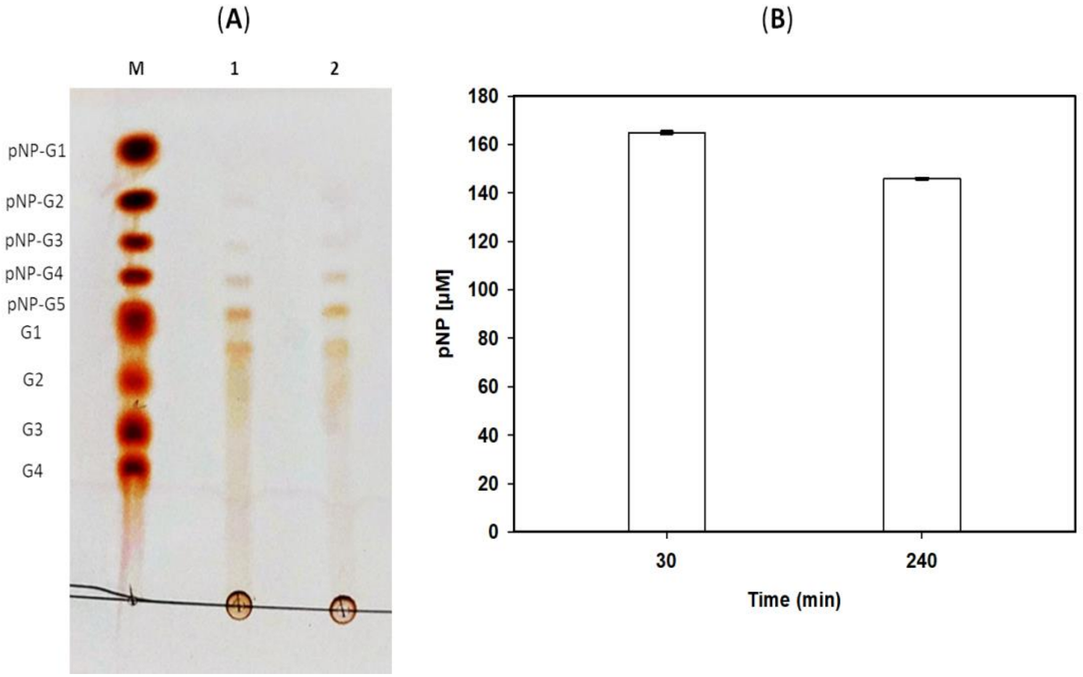

Figure S4. Analysis of the physical stability of cellulose-pNP in 50 mM sodium acetate buffer, pH 5.0. The reaction supernatants were analyzed by (**A**) TLC, and separately hydrolyzed by β-glucosidase to measure (**B**) the total pNP content. Cellulose-pNP (1.5 mg/mL) was incubated without enzyme and the supernatants (30 µL) were concentrated and loaded to TLC. Lane M shows the oligosaccharide standard mixture, while lanes 1 and 2 represent soluble reaction products at 0.5 and 4 h, respectively. The pNP content (n=2) at these time points was averaged and used to correct the total pNP release in the enzymatic reactions.

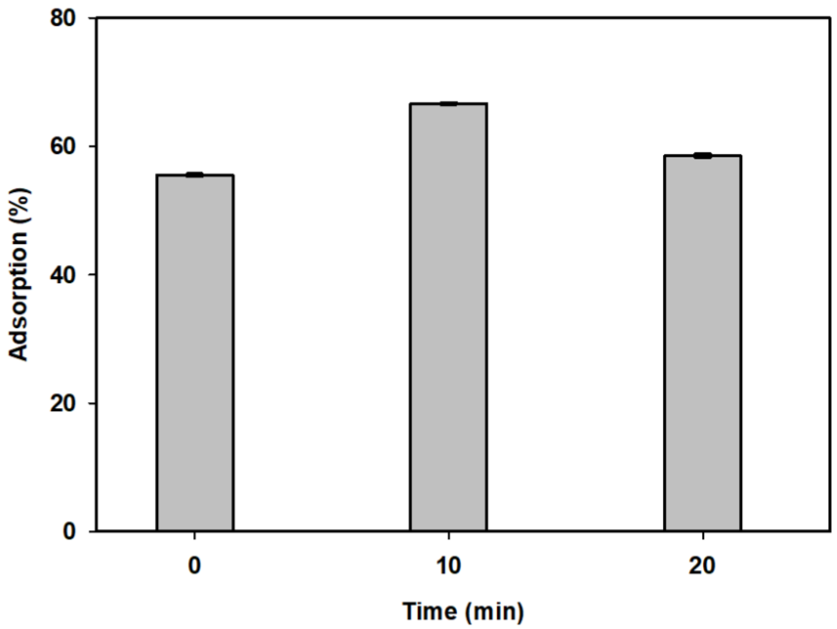

Figure S5. *T. reesei* cellulases adsorption on cellulose-pNP quantified by the Bradford assay. To minimize the hydrolysis and promote adsorption, cellulases (20 µg/mL) were incubated with cellulose-pNP (3.0 mg/mL) at a reduced incubation temperature (25 °C) under shaking (800 rpm). At certain times, a homogeneous sample (150 µL) was withdrawn and centrifuged (12400 g for 5 min at 4 °C). Soluble protein was measured using Roti-Nanoquant assay calibrated with BSA. Adsorbed protein (%) was determined from the difference in protein concentration before and after the incubation relative to the total protein concentration. Error bars show the S.D. (n=2).

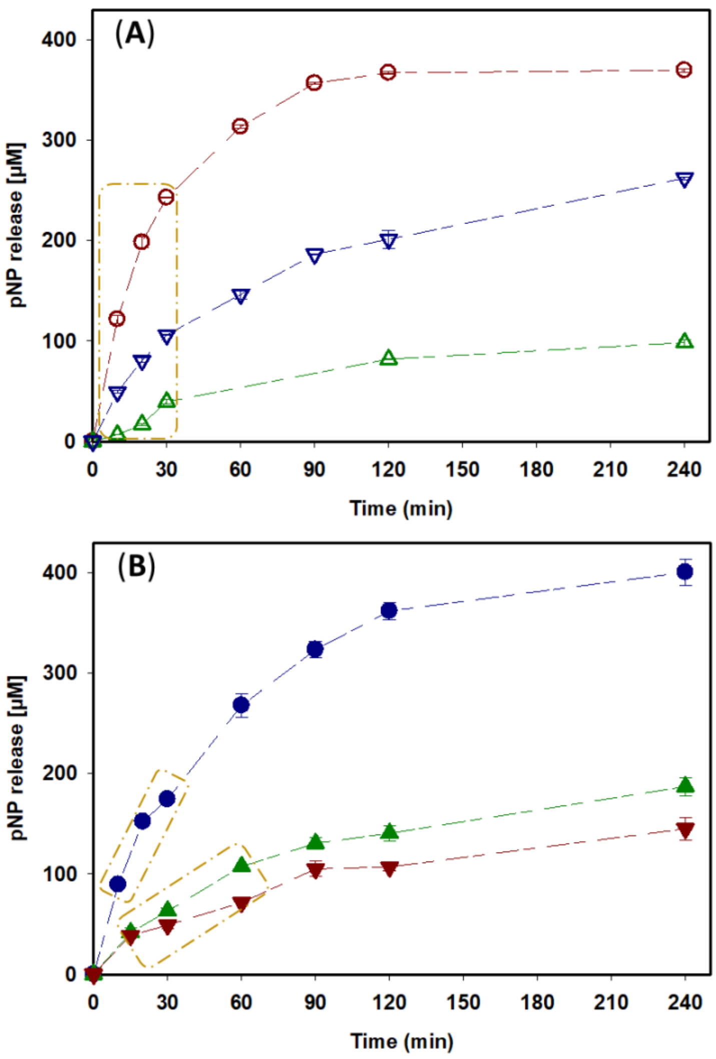

Figure S6. Time courses of cellulose-pNP hydrolysis by different hydrolytic enzymes. Hydrolysis was performed by (**A**) individual cellulases; *Tl*Cel7B (**-O-**), *Tr*Cel7A (**-Δ-**), and *Tr*Cel6A (**-∇-**) and (**B**) multi cellulases systems; cellulases mixture (**-•-**), cellulosome (**-▲-**), and disassembled cellulosome (**-▼-**). Degradation reactions (n=3) were performed by incubating cellulose-pNP (1.5 mg/mL) with different enzymes (1.0 µg/mL) under optimal conditions (see section 2.7 for details). Initial time points used to calculate the specific activities are highlighted.

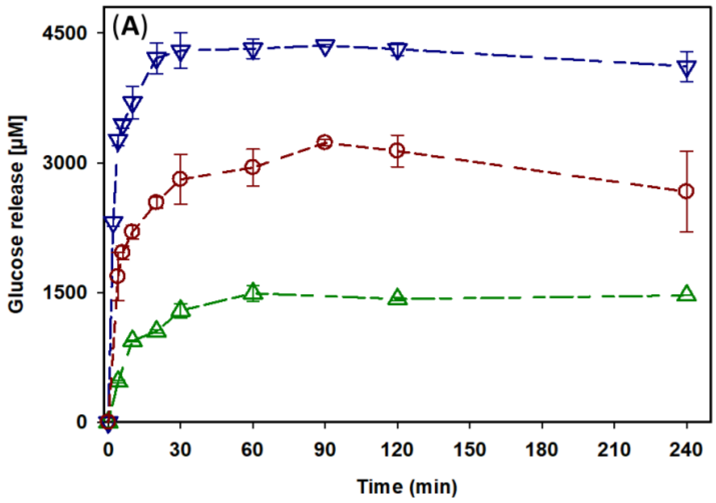

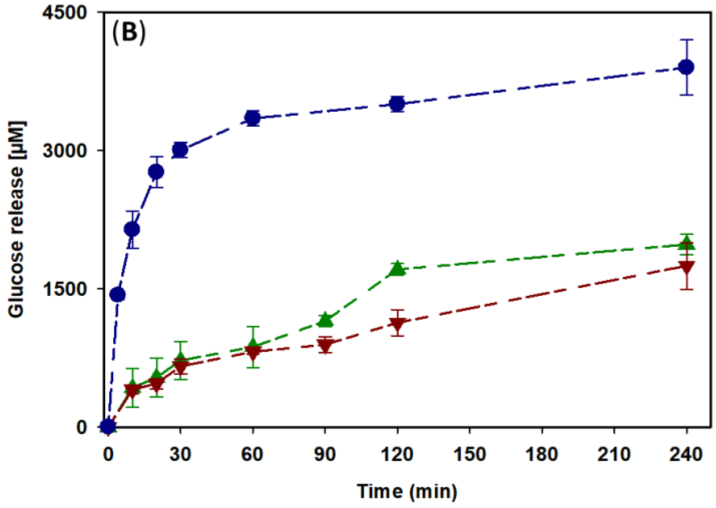

Figure S7. Time courses of unlabeled cellulose hydrolysis by different hydrolytic enzymes. Hydrolysis was performed by (**A**) individual cellulases; *Tl*Cel7B (**-O-**), *Tr*Cel7A (**-Δ-**), and *Tr*Cel6A (**-▽-**) and (**B**) multi cellulases systems; cellulases mixture (**-●-**), cellulosome (**-▲-**), and disassembled cellulosome (**-▼-**). Reaction conditions are similar to the cellulose-pNP hydrolysis (Fig. S6).

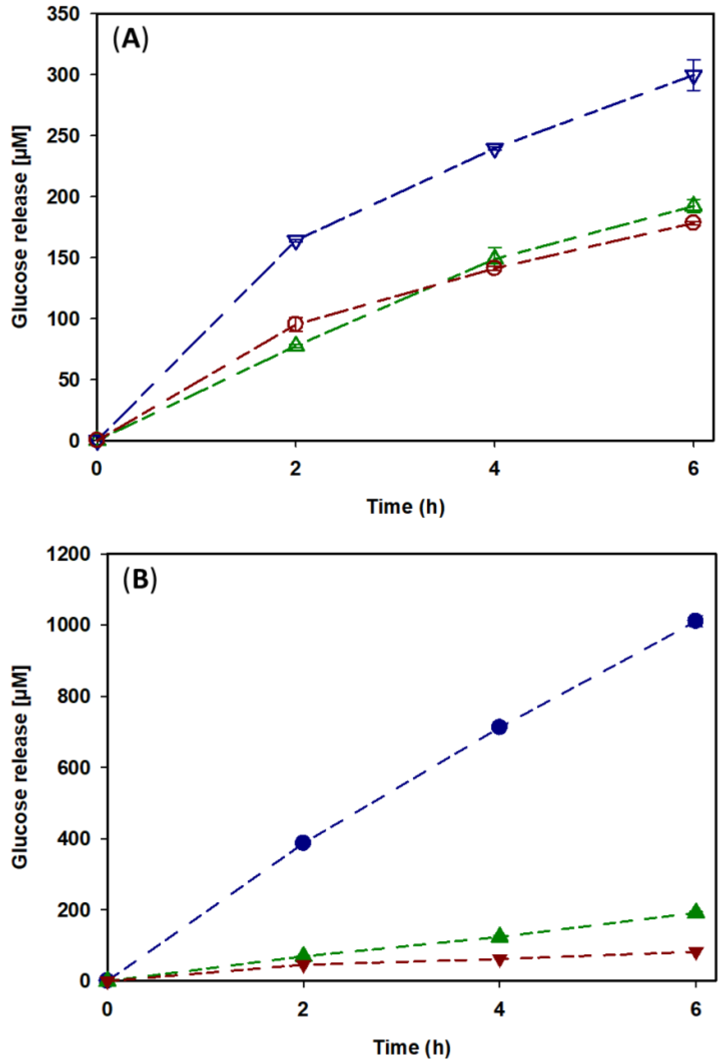

Figure S8. Time courses of Avicel hydrolysis by different hydrolytic enzymes. Hydrolysis was performed by (**A**) individual cellulases; *Tl*Cel7B (**-O-**), *Tr*Cel7A (**-Δ-**), and *Tr*Cel6A (**-▽-**) and (**B**) multi cellulases systems; cellulases mixture (**-•-**), cellulosome (**-▲-**), and disassembled cellulosome (**-▼-**). Degradation reactions (n=3) were performed by incubating Avicel (1.5 mg/mL) with either *Tl*Cel7B, *Tr*Cel7A, and *Tr*Cel6A at 10.0 µg/mL, or with cellulases mixture, cellulosome and disassembled cellulosome at 5.0 µg/mL. All reactions were supplemented with 2.0 µg/mL β-glucosidase to release the total glucose.

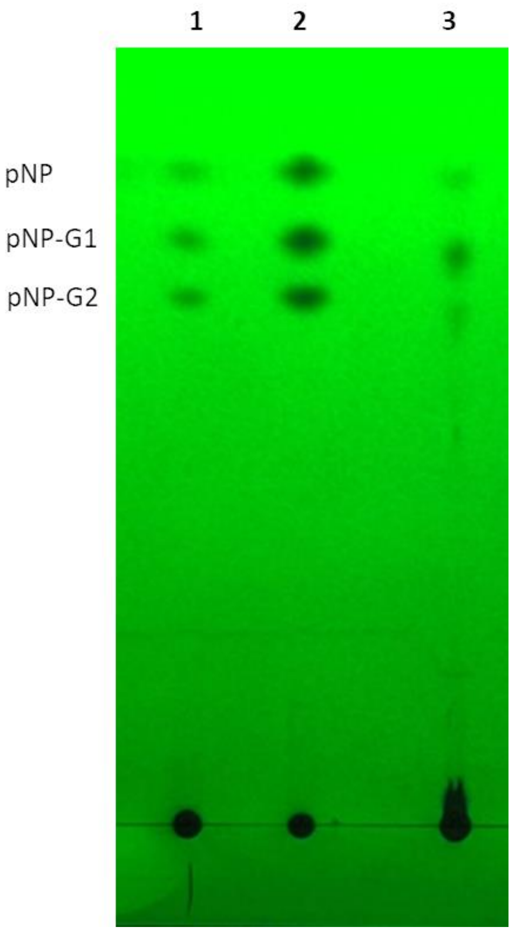

Figure S9. TLC analysis of soluble products used to estimate the primary cleavage action of *Tr*Cel7A on cellulose-pNP. Lane 1 and 2 contain standard mixtures of pNP, pNP-G1, and pNP-G2 at 1 mM and 2 mM, respectively. Lane 3 contains the supernatant from the *Tr*Cel7A reaction after 4 h of incubation. A total of 45 µL of reaction supernatant was concentrated by heating to a final volume of 5 µL prior to spotting on the TLC plate. All samples (lanes 1–3) were acidified with 50 µM HCl before spotting. TLC plates were visualized under UV light at 254 nm. Semi-quantitative densitometric analysis of the TLC spots using ImageJ indicated that the soluble pNP-labeled products comprised 24% pNP, 59% pNP-G1, and 17% pNP-G2.

Table S1. Rf values of different compounds present in the reference standard mixture used in TLCs.

**Compounds**	**Rf values***
pNP-G	0.85
pNP-G2	0.75
pNP-G3	0.67
pNP-G4	0.6
pNP-G5	0.54
G1	0.52
G2	0.42
G3	0.33
G4	0.25

*Rf values of individual compounds in the standards mixture (oligosaccharides G1 – G4 and soluble pNP-oligosaccharides pNP-G1 to pNP-G5) were calculated as distance travelled by compound/ total solvent front.

Table S2. Representative set of enzymes used for hydrolysis of cellulose-pNP, unlabeled synthetic cellulose and Avicel.

**Enzymes**	**Source**	**Preparation and characteristics**
Endo-glucancase (*Trichoderma longibrachiatum; Tl*Cel7B)	Commercial	Purified aqueous form, stock 13.8 mg/mL, 0.04 U/mg activity on Avicel
Cellobiohydrolase I (*Trichoderma reesei; Tr*Cel7A)	Produced from *T. reesei*	Purified aqueous form, stock 0.55 mg/mL, 0.05 U/mg activity on Avicel
Cellobiohydrolase II (*T. reesei; Tr*Cel6A, UniProtKB P07987)	Overexpressed in *Komagataella phaffii*	Lyophilized form, rebuffered stock 0.33 mg/mL, 0.06 U/mg activity on Avicel
Cellulases	Produced from *T. reesei*	Clarified aqueous form, stock 0.75 mg/mL, 0.52 U/mg activity on Avicel
Cellulosome	Puroduced from *Clostridium thermocellum*	Purified aqueous form, stock 0.15 mg/mL, 0.1 U/mg activity on Avicel

Supporting movie captions.

Movie S1. Time-lapse observation of *Tr*Cel7A acting on cellulose-pNP aggregates. Amplitude images (edge-sensitive) are shown to facilitate visualization. In total, 60 µL *Tr*Cel7A (100 µg/mL) were added and the experiment was recorded at room temperature in 50 mM sodium acetate buffer, pH 5.0. Streak-like artifacts originate from mobile sheets that were displaced by the AFM tip. The image acquisition rate and resolution were 0.84 frames/min and 2 nm/pixel, respectively. Scale bar, time stamps, and false-color scale are included in the video.

Movie S2. Snapshots showing partially directional degradation by *Tr*Cel7A. The image sequences were extracted from Movie S1 and upscaled by a factor of 2. Scale bar, time stamps, and false-color scale are included in the video.

Movie S3. Real-time observation of *Tr*Cel7A acting on isolated cellulose-pNP sheets. Height images were used to construct the video. A single sheet (height ~5 nm, green arrow) can be seen with multiple smaller sheets stacked on top (magenta arrow). Spherical structures (example indicated by a blue arrow) most likely correspond to unordered pNP-cellulose. In total, 40 µL *Tr*Cel7A (50 µg/mL) were added and the experiment was recorded at room temperature in 50 mM sodium acetate buffer, pH 5.0. The image acquisition rate and resolution were 5 frames/min and 1 nm/pixel, respectively. Scale bar, time stamps, and false-color scale are included in the video.
